# Broad-spectrum antiviral activity of the sigma-1 receptor antagonist PB28 against coronaviruses

**DOI:** 10.3389/fmicb.2025.1636035

**Published:** 2025-08-12

**Authors:** Gaojie Song, Lingling Cheng, Xingpeng Dong, Dapeng Li, Jia Cheng, Chao Shang, Xiao Li, Ran Zhu, Cuiling Zhang, Junwei Li

**Affiliations:** ^1^Jiangxi Provincial Key Laboratory of Cell Precision Therapy, School of Basic Medical Sciences, Jiujiang University, Jiujiang, China; ^2^The Second Affiliated Hospital of Jiujiang University, Jiujiang, China; ^3^School of Pharmacy, Nantong University, Nantong, China; ^4^Department of Neurosurgery, The First Hospital of Jilin University, Changchun, China; ^5^Changchun Veterinary Research Institute, Chinese Academy of Agricultural Sciences, Changchun, China; ^6^College of Medicine, Yanbian University, Yanji, China; ^7^Department of Infectious Disease, The Second Hospital of Nanjing, Affiliated to Nanjing University of Chinese Medicine, Nanjing, China

**Keywords:** coronavirus, PB28, sigma-1 receptor, antiviral activity, animal models

## Abstract

The continuous evolution of coronaviruses poses persistent and severe threats to both human and animal health. While α- and β-coronaviruses mainly infect mammals, including humans, γ-coronaviruses predominantly infect poultry, causing substantial economic losses. Their rapid mutation rates and wide host tropism underscore the urgent demand for pan-coronavirus therapeutics. Here, we systematically investigated the antiviral potency and mechanism of action of PB28, a selective sigma-1 receptor antagonist, across α-, β-, and γ-coronaviruses. Molecular docking predicted a stable interaction between PB28 and the sigma-1 receptor. PB28 exhibits robust *in vitro* antiviral activity, effectively inhibiting the replication of β-coronaviruses (SARS-CoV-2 and its Beta, Delta, and Omicron variants; HCoV-OC43), α-coronaviruses (PEDV and TGEV), and γ-coronaviruses (IBV). Broad-spectrum antiviral efficacy is further validated by viral titration assays. *In vivo*, PB28 administration in K18-hACE2 mice infected with SARS-CoV-2 Delta and BALB/c mice infected with HCoV-OC43 led to significantly reduced viral loads, attenuated multi-organ pathology, and improved survival and body weight maintenance. In parallel, PB28 treatment in IBV-infected chicken embryos and neonatal chicks enhanced survival, supported embryogenesis, and alleviated tissue damage. Collectively, PB28 demonstrates cross-genus antiviral efficacy, likely mediated through modulation of the sigma-1 receptor. These findings highlight PB28 as a promising lead compound for the development of pan-coronavirus therapeutics.

## 1 Introduction

Coronaviruses (CoVs) are enveloped positive-sense single-stranded RNA viruses belonging to the Coronaviridae family and Orthocoronavirinae subfamily and are characterized by large RNA genomes ranging from 27 to 31.5 kb (Shang et al., 2021). CoVs are taxonomically classified into four genera (α, β, γ, and δ coronaviruses) based on their genomic and serological characteristics (Wang et al., 2019; Lin et al., 2021). These viruses exhibit a broad host range and species-specific tropism (Tang et al., 2021), often resulting in respiratory, gastrointestinal, or systemic disease (Zhai et al., 2021). Alpha and beta coronaviruses primarily infect mammals including humans and pigs, while gamma coronaviruses predominantly infect birds. In contrast, delta coronaviruses are capable of crossing species barriers and infecting both birds and mammals (Woo et al., 2012), thereby increasing the risk of interspecies transmission.

Over the past twenty years three highly virulent human β-coronaviruses SARS-CoV, MERS-CoV, and SARS-CoV-2 have emerged, posing major threats to global health (Xiong et al., 2022). The global COVID-19 pandemic caused by SARS-CoV-2 has led to widespread morbidity and mortality across continents. Continuous mutations in the spike protein receptor-binding domain have led to variants such as Alpha, Beta, Delta, and Omicron that show increased immune evasion and reduced vaccine efficacy (He et al., 2021; Huang et al., 2022). In veterinary medicine animal coronaviruses remain major concerns. For example, Porcine epidemic diarrhea virus PEDV and transmissible gastroenteritis virus TGEV both classified as *α* coronaviruses cause lethal diarrhea in piglets (Turlewicz-Podbielska and Pomorska-Mól, 2021). The lack of cross-neutralization limits effective control of PEDV and TGEV (Lin et al., 2015). Additionally, avian infectious bronchitis virus IBV a *γ* coronavirus continues to threaten the poultry industry due to high serotype diversity and limited vaccine efficacy (Song et al., 2024). These challenges underscore the urgent need for effective safe and broad-spectrum antiviral agents.

The sigma-1 receptor is an intracellular chaperone protein involved in cellular stress responses, calcium homeostasis, and neuroprotection (Aishwarya et al., 2021). Recent studies have shown that SARS-CoV-2 non-structural protein 6 (NSP6) interacts directly with the sigma-1 receptor (Gordon et al., 2020b), modulating autophagy by restricting autophagosome expansion and inhibiting their fusion with lysosomes (Zhang et al., 2024). Our study showed that genetic deletion of the sigma-1 receptor restored autophagic flux disrupted by NSP6 and significantly reduced SARS-CoV-2 replication, highlighting its therapeutic potential (Zhang et al., 2024). PB28 is a small-molecule antagonist of the sigma-1 receptor that disrupts virus-host protein interactions, activates apoptotic signaling, and exhibits antitumor properties in A549 cells (Abate et al., 2020). However, the antiviral spectrum of PB28 across multiple coronavirus genera remains incompletely characterized. To investigate this, we developed a cross-species infection platform covering α-, β-, and γ-coronaviruses and systematically evaluated the antiviral efficacy and mechanisms of PB28, generating evidence to support host-directed pan-coronavirus strategies. Host-directed therapy (HDT) is increasingly recognized as a promising approach for broad-spectrum antiviral drug development, largely because it minimizes the likelihood of resistance by targeting host factors (Shi et al., 2023). Overall, PB28 displays potent and broad-spectrum antiviral activity against α-, β-, and γ-coronaviruses by targeting virus-host interactions through selective sigma-1 receptor antagonism. By modulating conserved host pathways rather than virus-specific proteins, PB28 offers a strategic advantage in overcoming viral mutation and immune evasion. These findings support the development of PB28 as a pan-coronavirus therapeutic, particularly suited to combat emerging variants and cross-species transmission.

## 2 Materials and methods

### 2.1 Cell lines and viral strains

African green monkey kidney (Vero E6, CL-0491), swine testicle (ST, CL-0219), and human ileocecal adenocarcinoma (HCT-8, CL-0098) cell lines were obtained from our laboratory stocks, originally purchased from Procell Life Science & Technology Co., Ltd. (Wuhan, China). The cell lines were selected based on viral genus-specific tropism. Vero E6 cells were used for the propagation of SARS-CoV-2, its variants, PEDV, and IBV. HCT-8 cells were used for HCoV-OC43, and ST cells for TGEV. A panel of viral strains was used in this study, including SARS-CoV-2 (IME-BJ01 strain, GenBank: MT291831), Omicron variants BA.1 (CoV/human/CHN_CVRI-01/2022) and BA.2 (CoV/human/CHN_CVRI-12/2022), porcine epidemic diarrhea virus (PEDV, GenBank: OM814174), transmissible gastroenteritis virus (TGEV, GenBank: FJ755618), human coronavirus OC43 (HCoV-OC43, VR-1558), Delta variant (CSTR.16698.06. NPRC6. CCPM-B-V-049-2105-6), Beta variant (CSTR: 16698.06. NPRC2.062100001), and infectious bronchitis virus (IBV, AV1511). All viral strains were provided by our laboratory. Experiments involving live SARS-CoV-2 were conducted in BSL-3 containment laboratories.

### 2.2 Experimental animals and embryonated eggs

Female BALB/c mice, 4 weeks old and specific pathogen-free (SPF), were obtained from Beijing Sipeifu Biotechnology. K18-hACE2 transgenic female mice, aged 8 to 10 weeks and maintained under SPF conditions, were supplied by the National Institute for Food and Drug Control. White Leghorn embryonated eggs were obtained from Boehringer Ingelheim Viton Biotechnology Co., Ltd. (Beijing, China), and the hatched chicks were used for subsequent experiments.

### 2.3 Reagents and materials

PB28 dihydrochloride (Cat. No. 172907–03-8) was sourced from MedChemExpress (United States). Ribavirin injection (Batch No. H20013147) was obtained from Chia Tai Tianqing Pharmaceutical Group Co., Ltd. Arbidol hydrochloride (ARB) was supplied by CSPC Ouyi Pharmaceutical Co., Ltd. Remdesivir (Cat. No. HY-104077) was obtained from MedChemExpress (United States). Dimethyl sulfoxide (DMSO, Batch No. D4540) and TRIzol reagent (Batch No. 15596018) were purchased from Sigma-Aldrich and Invitrogen, respectively. Paraformaldehyde solution (4%, Batch No. P0099-500 mL) was obtained from Beyotime Biotechnology.

### 2.4 Molecular docking analysis

Docking simulations were conducted with AutoDock Vina 1.1.2, an open-source platform for virtual screening and ligand-receptor docking. This software exhibits enhanced precision in predicting binding conformations compared with AutoDock 4 (Trott and Olson, 2010). SDF files of the candidate compounds were retrieved from the PubChem database and then converted into MOL2 format using Open Babel 2.4.1. The crystallographic structure of Sigma-1 receptor (PDB ID: 5HK1) was obtained from the Protein Data Bank. Ligands and receptors were prepared following the standard AutoDock Vina 1.1.2 workflow. All water molecules were eliminated, non-polar hydrogens were added, and Gasteiger charges were computed. The prepared files were saved in PDBQT format. Lower docking scores reflect stronger ligand-receptor affinity. The docking pose with the best binding energy was selected as the optimal complex, and molecular interactions were visualized using PLIP (Salentin et al., 2015).

### 2.5 Cell viability assay

Vero E6, HCT-8, and ST cells were seeded in 96-well plates at a density of 5 × 10^3^ cells per well and incubated overnight to allow cell adherence. PB28 was serially diluted in complete culture medium to achieve final concentrations of 0.625, 1.25, 2.5, and 5 μM. A volume of 100 μL from each dilution was added to triplicate wells. Plates were incubated at 37°C in a humidified 5% CO₂ atmosphere. Wells containing medium alone served as blank controls. After 48 h of treatment, 10 μL of CCK-8 solution and 90 μL of fresh medium were added to each well and incubated for 30 min. Absorbance at 450 nm was recorded using a microplate reader to assess cell viability.

### 2.6 Viral titer determination

Vero E6, ST, and HCT-8 cells were plated in 6-well plates at a density of 2 × 10^5^ cells per well and incubated overnight for cell attachment. Cells were allocated into control, model, and PB28 treatment groups (0.625, 1.25, 2.5, and 5 μM). Except for control wells, all groups were exposed to viral infections: Vero E6 cells were infected with SARS-CoV-2 and its variants (MOI = 0.08), PEDV (MOI = 3.89 × 10^−3^), or IBV (MOI = 0.1); ST cells were infected with TGEV (MOI = 1.0 × 10^−4^), and HCT-8 cells were infected with HCoV-OC43 (MOI = 1.0). After 2 h of viral adsorption, the inoculum was removed, and PB28 was added at the indicated concentrations. Cells were then incubated for 48 h. Cells and supernatants were harvested for downstream analysis. For TCID_50_ assays, 96-well plates were prepared with 5 × 10^3^ cells per well via overnight incubation. Viral supernatants were subjected to 10-fold serial dilutions in culture medium, and 100 μL was added to each well. Uninfected wells served as negative controls. After 2 h of incubation, 100 μL of fresh growth medium was added to each well. Plates were incubated under standard conditions, and TCID_50_ values were calculated using the Kärber method.

### 2.7 Protective effect of PB28 against Delta variant

The SARS-CoV-2 Delta variant, characterized by increased transmissibility and virulence compared to earlier strains, caused significant surges in global hospitalizations and mortality during 2021 (Mlcochova et al., 2021). Due to its rapid replication and notable immune evasion capabilities, the Delta variant has frequently been utilized in preclinical studies of antiviral efficacy (Saito et al., 2022). Transgenic K18-hACE2 mice were used as an *in vivo* model for Delta variant infection and were randomly divided into five groups (n = 10 per group): a mock group, a virus-infected model group, PB28-treated groups (3.3, 10, and 30 mg/kg), and a Remdesivir-treated group (50 mg/kg). Except for the mock group, all mice were anesthetized via intraperitoneal injection of pentobarbital sodium (30 mg/kg) and inoculated intratracheally with 30 μL of Delta variant suspension containing 10^4^ TCID₅₀ per mouse (Shang et al., 2021). PB28 was administered via oral gavage, while Remdesivir was given intraperitoneally once daily for seven consecutive days. The mock and model groups received equal volumes of saline. On day 3 post-infection, three mice per group were randomly selected, anesthetized with 2% isoflurane, euthanized via cervical dislocation, and heart, brain, lung, and nasal turbinate tissues were collected for viral titer quantification, histopathological analysis (H&E staining), and RT-qPCR assessment of E and N gene expression. The remaining mice were monitored daily for survival and body weight until day 7 post-infection (dpi).

### 2.8 Histopathological analysis

Histological examinations were performed as previously described (Song et al., 2022). The trachea, lungs, and kidneys were rinsed with PBS and fixed in 0.4% paraformaldehyde for 48 h. Fixed tissues were embedded in paraffin, sectioned, and stained with H&E. Sections were examined microscopically, and histological alterations were scored according to a revised evaluation system (Song et al., 2024). Scoring criteria were: 0 = no lesion; 1 = affected area <10%; 2 = 10–50%; 3 = 50–90%; 4 = >90%. Pathological scores for the trachea, lungs, brain, and kidneys were statistically analyzed.

### 2.9 Protective effect of PB28 against HCoV-OC43

To evaluate the *in vivo* antiviral efficacy of PB28, a BALB/c mouse model of HCoV-OC43 infection was employed, which is widely used for studying *β*-coronavirus pathogenesis and treatment responses (Shen et al., 2019; Girkin et al., 2023). ARB, a broad-spectrum antiviral agent with validated activity against HCoV-OC43, was used as a positive control owing to its ability to inhibit viral entry and replication (Zhu et al., 2020). Female BALB/c mice (4 weeks old) were anesthetized with 2% isoflurane and intranasally inoculated with 50 μL of HCoV-OC43 suspension containing 400 PFU. Twelve hours post-infection, mice were randomly assigned to four groups: a virus-infected model group receiving intraperitoneal PBS, three PB28-treated groups (3.3, 10, and 30 mg/kg), and a group receiving 25 mg/kg ARB as a positive control (Xie et al., 2022). All treatments were administered once daily for seven consecutive days. On day 3 post-infection, three mice from each group were euthanized under deep anesthesia for tissue collection (heart, brain, and lungs), which were used for viral load quantification and H&E staining. Body weight and survival were monitored daily throughout the study.

### 2.10 Protective effect of PB28 against IBV infection in embryonated eggs

Fertilized chicken eggs were randomly assigned to five experimental groups (*n* = 10 per group), including a control group, a virus-infected model group, three PB28 treatment groups (0.08, 0.16, and 0.32 mg/kg), and a ribavirin group (18 mg/kg). All groups, except the controls, were inoculated with 100 EID₅₀ of IBV via the allantoic cavity. Prior to inoculation, viral suspensions were pre-incubated with each test compound for 30 min. At the end of the experiment, embryonic viability was recorded. Embryo weight and length were measured, and pathological changes were evaluated via visual inspection and photographic documentation. Scores from 1 to 4 were assigned to indicate normal development, reduced weight, visible malformation, and death, respectively.

### 2.11 Protective effect of PB28 against IBV infection in chicks

SPF White Leghorn chicks (1 day old) were randomly divided into six groups (*n* = 10 per group). To induce infection, the IBV-M41 strain (300 μL, 10^6.5^ EID₅₀) was administered through ocular and intranasal routes. Control chicks received an equal volume of virus-free allantoic fluid. Treatment groups received PB28 at doses of 4, 8, or 16 mg/kg, or ribavirin at 90 mg/kg. Clinical symptoms and mortality were monitored daily. On day 4 post-infection, three chicks from each group were randomly selected, deeply anesthetized via intraperitoneal injection of pentobarbital sodium (45 mg/kg), euthanized by cervical dislocation, and subjected to viral load quantification and histopathological analysis.

### 2.12 Statistical analysis

Data were analyzed statistically using GraphPad Prism 8.0. One-way ANOVA and t-tests were applied to assess intergroup differences. A *p*-value of <0.05 was considered statistically significant.

## 3 Results

### 3.1 Interaction mode of PB28 with sigma-1 receptor

**Figure 1:**
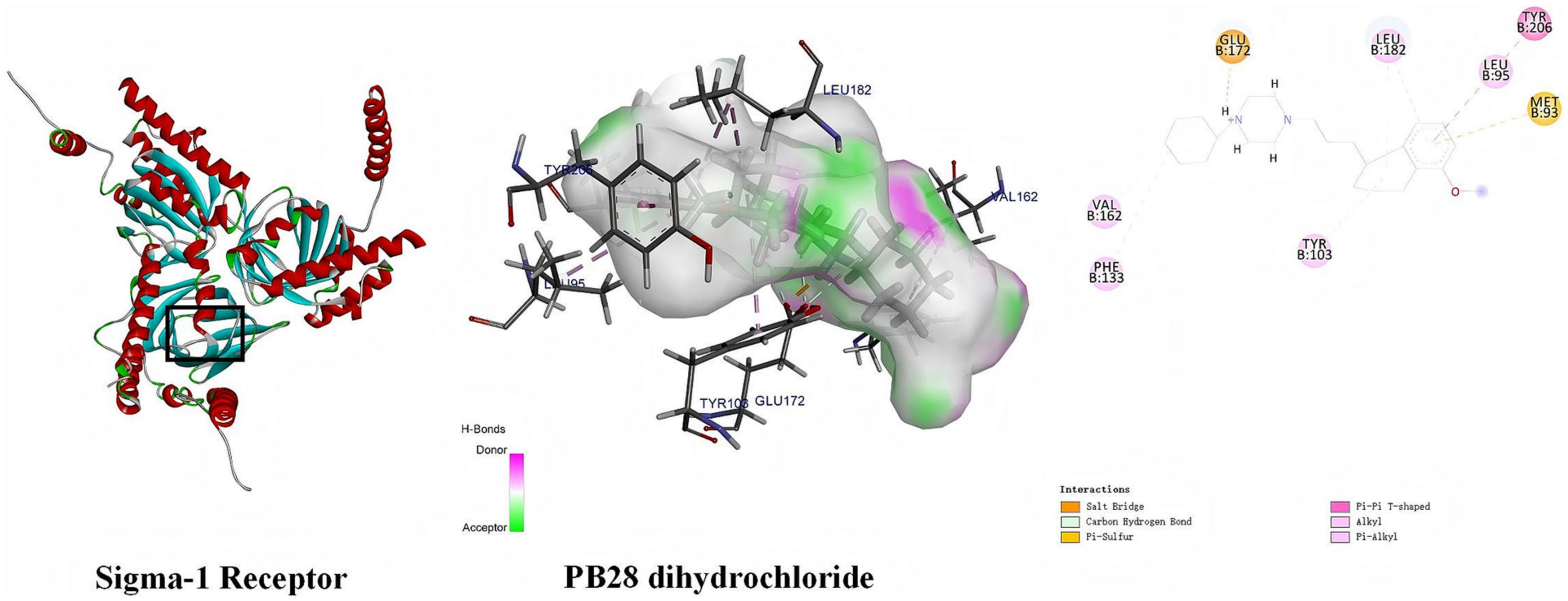
Binding interactions between the sigma-1 receptor and PB28 dihydrochloride, as predicted by molecular docking.

Molecular docking analysis was conducted to explore the binding interactions between PB28 and the sigma-1 receptor. A lower docking score generally reflects stronger ligand–receptor binding affinity. The calculated binding energy was −9.104 kcal/mol, and the interaction model between PB28 dihydrochloride and sigma-1 receptor is shown in Figure 1. Key interactions included a salt bridge (GLU172), *π*–sulfur interaction (MET93), π–π T-shaped interaction (TYR206), alkyl interactions (TYR103, PHE133, VAL162, LEU182), and π–alkyl interactions (LEU95, LEU182). These interactions collectively stabilized the protein’s tertiary structure through a network of non-covalent forces and contributed to its biological function. The results indicate that PB28 may modulate sigma-1 receptor activity.

### 3.2 Effects of PB28 on Vero E6, ST, and HCT-8 cell viability

**Figure 2:**
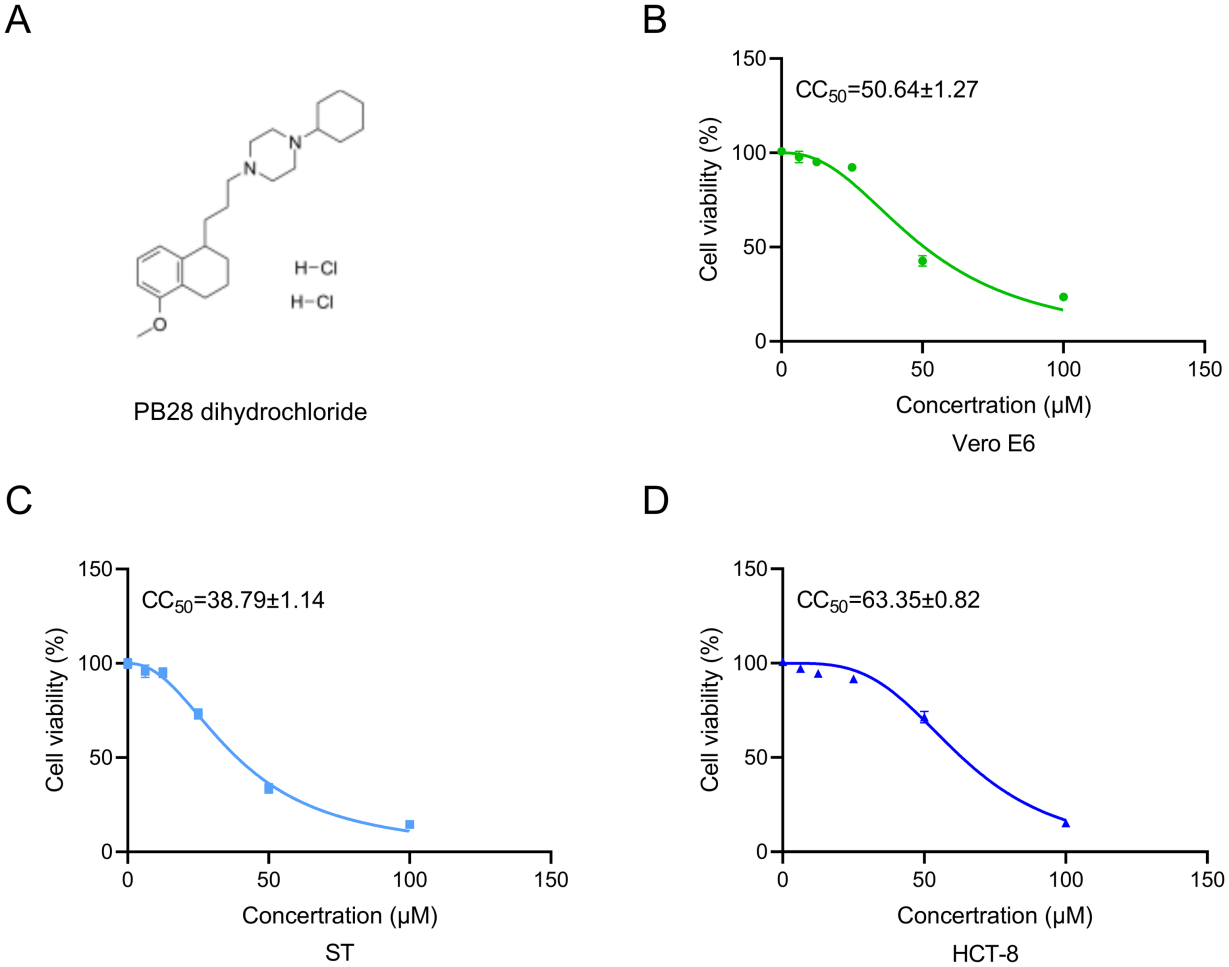
**(A)** Chemical structure of PB28 dihydrochloride. **(B–D)** PB28 effects on cell viability following 48-h exposure in Vero E6 **(B)**, ST **(C)**, and HCT-8 **(D)** cell lines (*n* = 3).

Figure 2A depicts the molecular structure of PB28 dihydrochloride. Cell viability was assessed using the CCK-8 assay following 48-h treatment of Vero E6, HCT-8, and ST cells with varying concentrations of PB28. Dose–response curves were modeled using non-linear regression (GraphPad Prism) to determine the CC_50_ values. The CC_50_ values for PB28 were determined to be 50.64 ± 1.27 μM for Vero E6 cells (Figure 2B), 38.79 ± 1.14 μM for ST cells (Figure 2C), and 63.35 ± 0.82 μM for HCT-8 cells (Figure 2D).

### 3.3 Inhibitory effects of PB28 on coronavirus replication in cells

**Figure 3:**
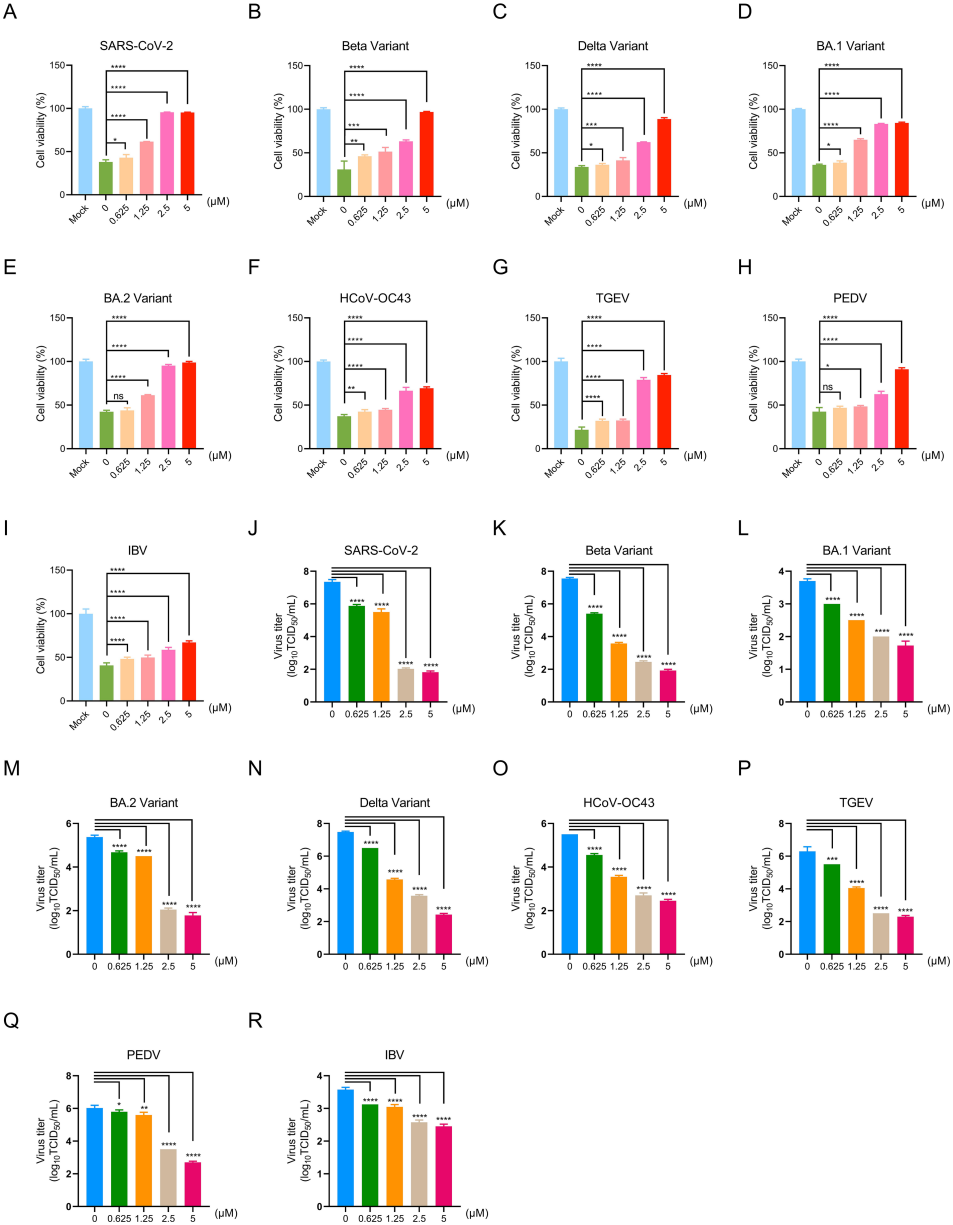
Antiviral effects of PB28 against coronaviruses in vitro. **(A–I)** PB28 treatment restores cell viability in infected cells (*n* = 3). **(J–R)** PB28 significantly reduces viral titers in cells infected with multiple coronavirus strains (*n* = 5). *p < 0.05, **p < 0.01, ***p < 0.001, ****p < 0.0001.

To comprehensively evaluate the antiviral activity of PB28 against SARS-CoV-2 and its potential broad-spectrum efficacy, we initially focused on the Betacoronavirus genus. In a Vero E6 cell model infected with the SARS-CoV-2 prototype strain, PB28 (0.625–5 μM) significantly improved cell viability in a dose-dependent manner, as determined by CCK-8 assays (Figure 3A). To further explore its pan-variant efficacy, we assessed PB28 against several key variants of concern (VOCs), including Beta, Delta, BA.1, and BA.2. Across all tested variant infection models, PB28 exhibited similar dose-dependent antiviral responses (Figures 3B–E), consistent with its activity against the ancestral strain. Importantly, PB28’s antiviral effects extended to other members of the Betacoronavirus genus. In HCT-8 cells infected with HCoV-OC43, PB28 also produced a notable dose-dependent inhibitory effect (Figure 3F). To assess its cross-genus antiviral spectrum, we examined PB28 activity in two Alphacoronavirus models: TGEV-infected ST cells (Figure 3G) and PEDV-infected Vero E6 cells (Figure 3H). In the IBV model, PB28 effectively alleviated virus-induced cytopathic effects in a concentration-dependent manner (Figure 3I).

To quantify PB28’s antiviral potency, half-maximal effective concentrations (EC_50_) were determined for multiple coronaviruses. In Vero E6 cells, PB28 demonstrated EC_50_ values ranging from 1.4 to 5.62 μM for SARS-CoV-2 and its variants, as well as PEDV and IBV (Supplementary Table 1). For TGEV and HCoV-OC43, the EC_50_ values in ST and HCT-8 cells were 2.01 ± 0.08 μM and 4.12 ± 0.22 μM, respectively. A selectivity index (SI) greater than 1 is generally indicative of antiviral efficacy, with higher SI values reflecting greater safety. PB28 exhibited SI values ranging from 9.01 to 36.17 (Supplementary Table 1), demonstrating both strong antiviral activity and favorable safety profiles across tested strains.

To investigate the anti-replication efficacy of PB28, viral titers were quantified by TCID_50_ assay. In the SARS-CoV-2-infected Vero E6 cells, the viral titer in the untreated group reached 7.35 ± 0.12 log_10_ TCID_50_/mL, whereas treatment with PB28 at 0.625, 1.25, 2.5, and 5 μM resulted in a dose-dependent reduction to 5.88 ± 0.08, 5.50 ± 0.18, 2.03 ± 0.05, and 1.83 ± 0.06 log_10_ TCID_50_/mL, respectively (Figure 3J), exhibiting a clear dose-dependent decrease. Similar dose-dependent reductions were observed for Beta, BA.1, BA.2, and Delta variants, with 5 μM PB28 reducing viral titers by approximately 5.63, 1.98, 3.60, and 5.05 log_10_ TCID_50_/mL, respectively, compared to the mock group (Figures 3K–N). In TGEV- and PEDV-infected systems, treatment with PB28 led to a reduction of approximately 4.0 and 3.33 log_10_ TCID_50_/mL, respectively (Figures 3P,Q). In HCoV-OC43 and IBV models, 5 μM PB28 treatment decreased viral titers by approximately 3.05 and 1.13 log_10_ TCID_50_/mL, respectively (Figures 3O,R). Meanwhile, SARS-CoV-2 replication was markedly inhibited in Vero E6 cells lacking the Sigma-1 receptor (Sigma-1R-KO). PB28 effectively reduced viral titers; however, its antiviral efficacy was not significantly different when used in combination with Sigma-1R knockout compared to Sigma-1R knockout alone (Supplementary Figure S1). Collectively, PB28 treatment significantly reduced viral titers in Vero E6, HCT-8, and ST cells, indicating its broad-spectrum inhibitory activity against α-, β-, and *γ*-coronaviruses.

### 3.4 PB28 protective effects against the SARS-CoV-2 Delta variant

**Figure 4:**
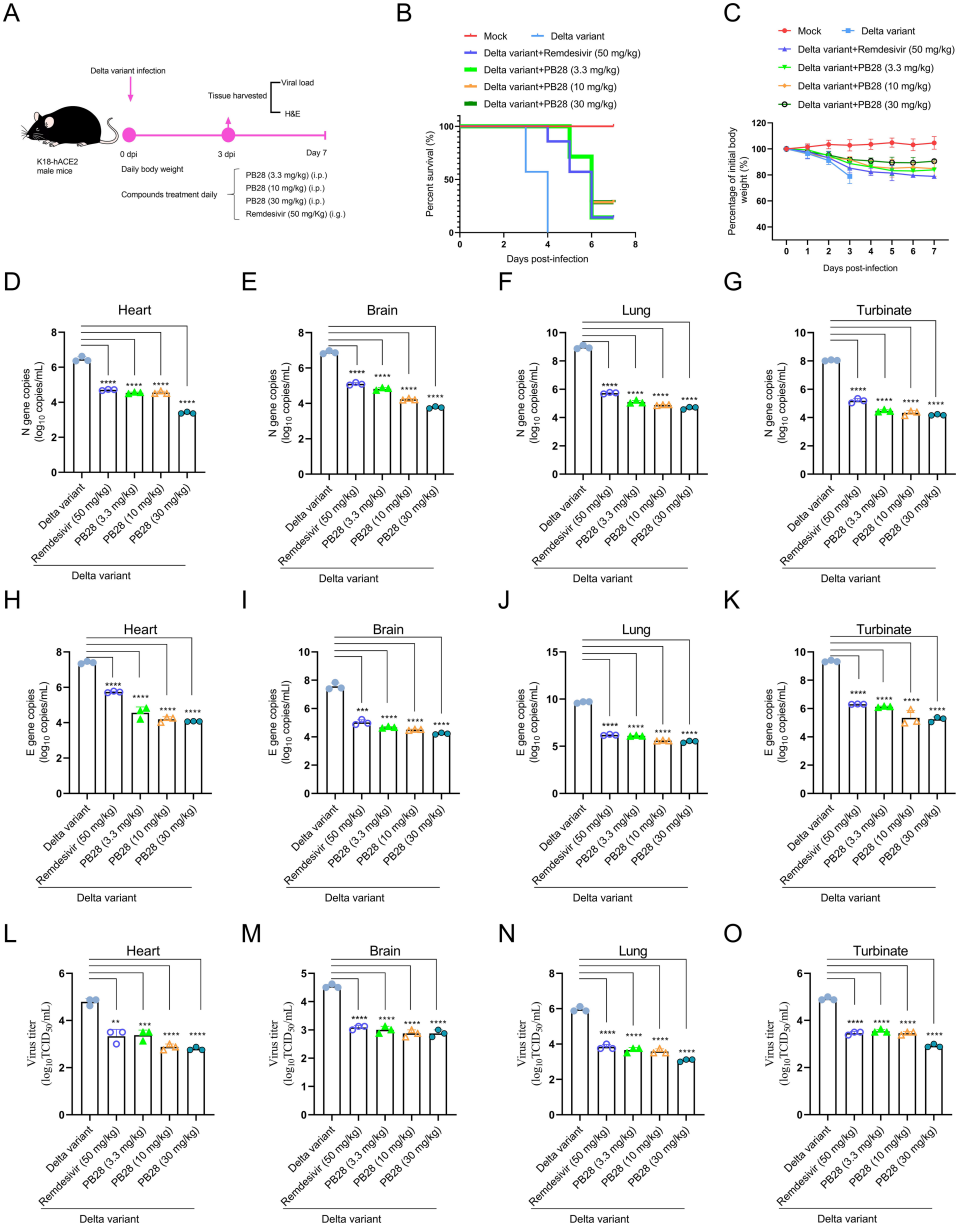
Therapeutic efficacy of PB28 in K18-hACE2 transgenic mice models challenged with Delta variant. **(A)** Schematic of viral challenge protocol timeline. **(B)** Survival outcomes for each group of mice. **(C)** Body weight changes over the course of the study. **(D–G)** Viral load assessments in the heart, brain, lungs, and nasal turbinates (*n* = 3). **(H–K)** Delta variant N gene by RT-qPCR (*n* = 3). **(L–O)** Delta variant E gene by RT-qPCR (*n* = 3). *p < 0.05, **p < 0.01, ***p < 0.001, ****p < 0.0001.

Building on PB28’s ex vivo antiviral activity, its therapeutic efficacy was assessed in K18-hACE2 transgenic mice infected intranasally with the SARS-CoV-2 Delta variant. The experimental design is presented in Figure 4A. As shown in Figure 4B, all untreated mice succumbed to infection at 4 dpi. In contrast, PB28- and Remdesivir-treated mice exhibited delayed mortality and increased survival, indicating therapeutic benefit. Body weight analysis showed marked weight loss in the Delta group, while treatment with PB28 or Remdesivir mitigated weight loss and promoted recovery (Figure 4C). RT-qPCR, targeting the N and E genes, was employed to quantify viral RNA in heart, brain, lungs, and nasal turbinates at 3 dpi. PB28 significantly reduced N and E gene copy numbers in all examined tissues (Figures 4D–K) and concomitantly lowered infectious viral titers (Figures 4L–O). H&E-stained sections (Supplementary Figure S2A) revealed widespread pulmonary necrosis, exudation, inflammatory infiltration, and alveolar wall thickening. Additionally, neuronal degeneration, brain nuclear pyknosis, and severe myocardial structural disruption were noted. Treatment with PB28 or Remdesivir ameliorated these lesions, reducing pulmonary inflammation, preserving alveolar structure, attenuating neuronal damage, and mitigating cardiac pathology. PB28 treatment significantly improved histopathology and reduced damage scores (Supplementary Figures S2B–D).

### 3.5 PB28-mediated protection against HCoV-OC43 in mice

**Figure 5:**
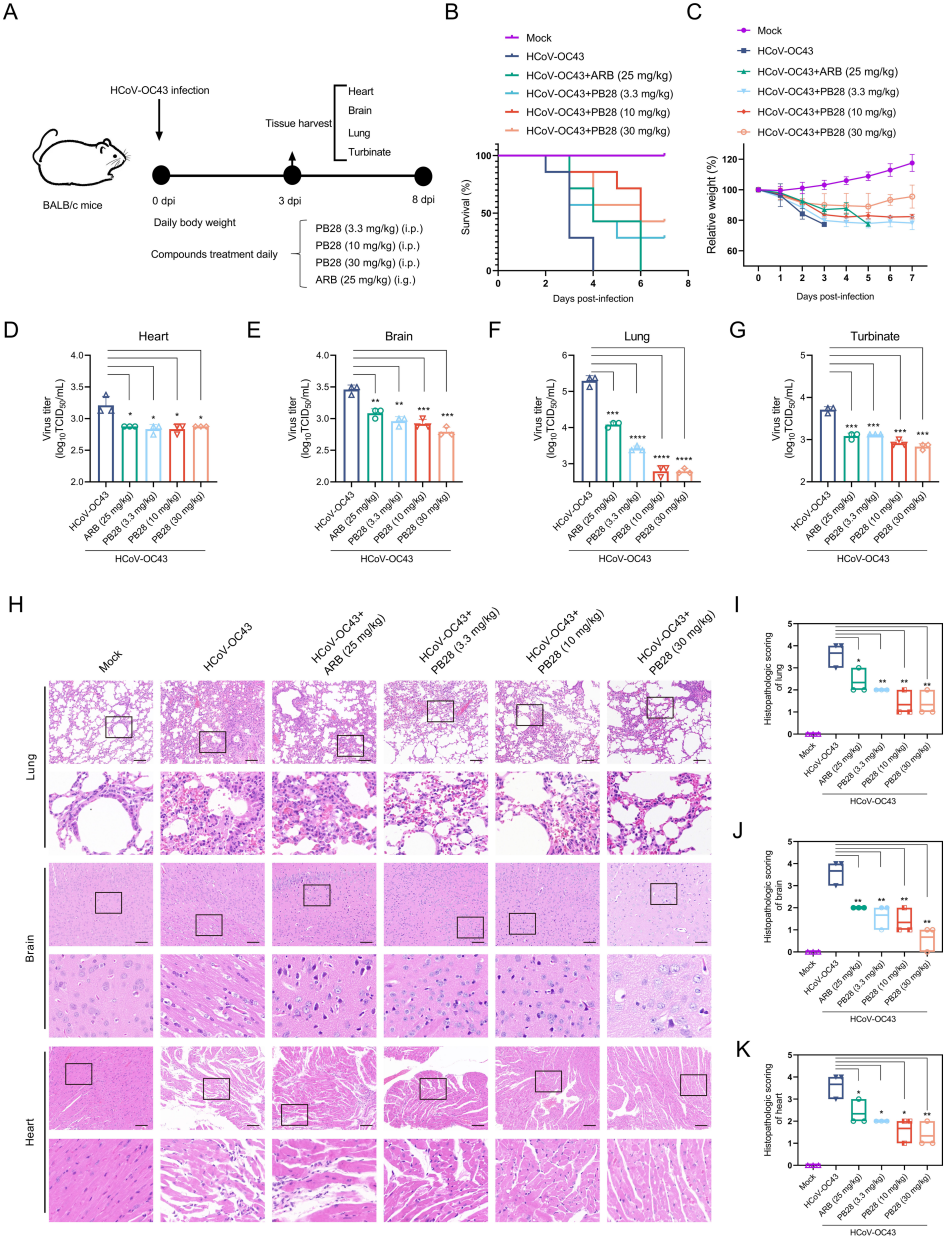
Protective efficacy of PB28 in BALB/c mice infected with HCoV-OC43. **(A)** Experimental design of HCoV-OC43 challenge. **(B)** Survival curves of infected mice (*n* = 7). **(C)** Body weight changes (*n* = 7). **(D–G)** Viral titers in heart, brain, lung, and turbinate tissues on day 3 post-infection (*n* = 3). **(H)** Histological alterations in lung, brain, and heart tissues as assessed by H&E staining (scale bar = 100 μm; *n* = 3). **(I–K)** Histopathological scores of corresponding tissues (*n* = 3). *p < 0.05, **p < 0.01, ***p < 0.001, ****p < 0.0001.

The experimental design is illustrated in Figure 5A. Mice in the HCoV-OC43 group began to die at 2 dpi, with complete mortality by 4 dpi. In contrast, PB28- and ARB-treated mice showed delayed mortality onset until 3 dpi. Notably, all ARB-treated mice died at 6 dpi, indicating that PB28 conferred superior survival benefits (Figure 5B). Mice in the HCoV-OC43 group exhibited significant weight loss compared to controls, which was alleviated by PB28 or ARB treatment (Figure 5C). At 3 dpi, viral loads in heart, brain, lungs, and nasal turbinates were quantified. PB28 significantly inhibited viral replication in these tissues, exhibiting efficacy comparable to that of ARB (Figures 5D–G). Histological examination revealed severe pulmonary lesions, including inflammatory infiltration, alveolar wall thickening and dilation, hemorrhage, and vascular necrosis. The myocardium exhibited atrophy, nuclear pyknosis, interstitial edema, and inflammatory infiltration. Mild neuronal degeneration and nuclear condensation were also evident in brain tissues (Figure 5H). PB28 treatment alleviated these histopathological changes and significantly lowered pathology scores (Figures 5I–K), indicating stronger protection than ARB.

### 3.6 PB28 exhibits antiviral efficacy against IBV in an embryonated egg model

**Figure 6:**
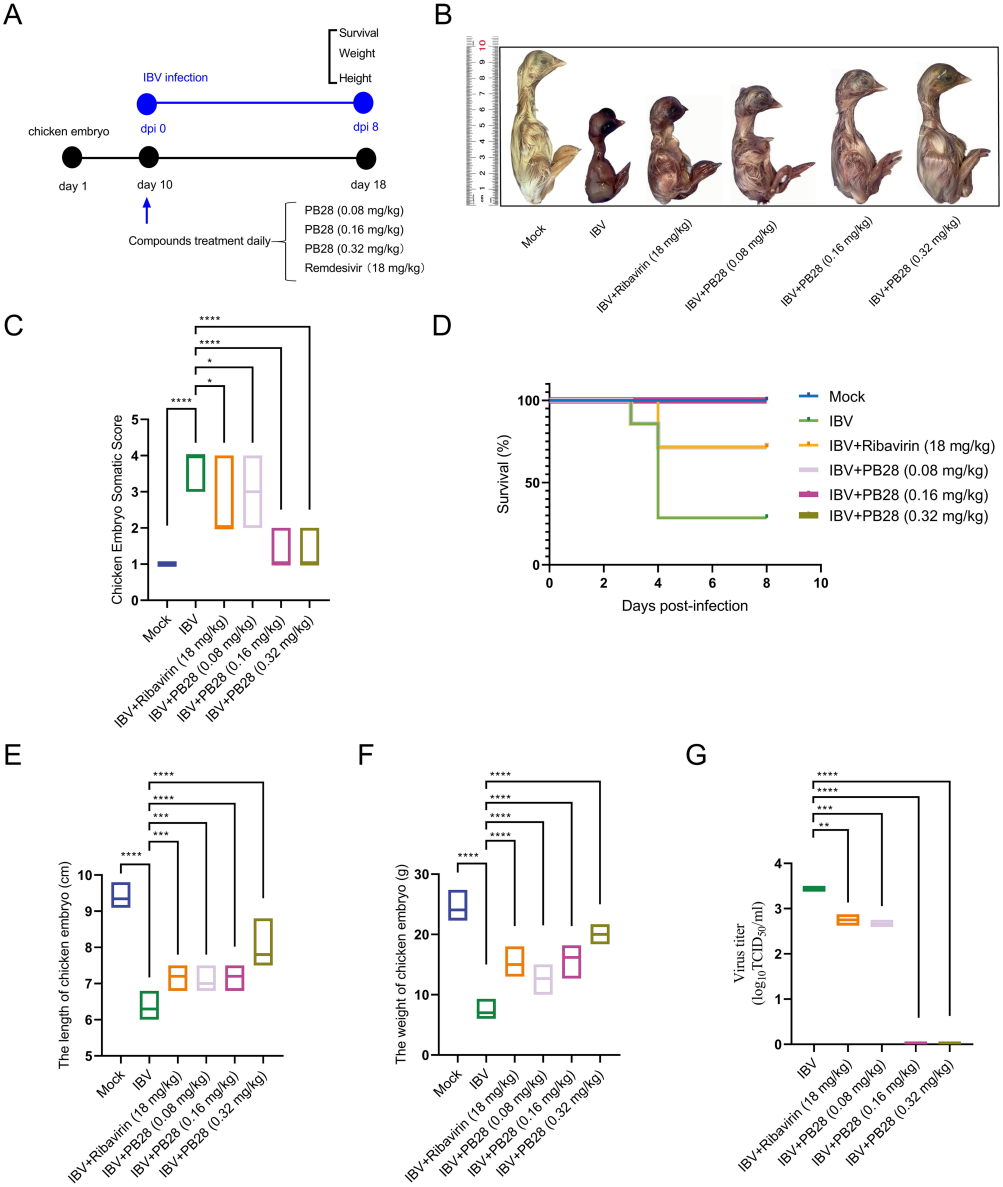
PB28 confers protective effects against IBV infection in embryonated chicken eggs. **(A)** Diagram illustrates the experimental protocol. **(B)** Representative photographs of embryos. **(C)** Pathological scores of embryonic lesions (*n* = 7). **(D)** Embryo survival rates (*n* = 7). **(E)** Embryo lengths (*n* = 7). **(F)** Embryo weights (*n* = 7). **(G)** Viral titers in embryonic tissues (*n* = 7). *p < 0.05, **p < 0.01, ***p < 0.001, ****p < 0.0001.

The protective efficacy of PB28 against IBV was evaluated in a chicken embryo infection model. The experimental protocol is depicted in Figure 6A. No embryo mortality occurred within 24 h across all groups. IBV-infected embryos exhibited severe developmental delay and body size reduction. In contrast, embryos treated with PB28 or ribavirin demonstrated improved morphology and significantly lower clinical scores (Figures 6B,C). No embryo mortality was observed in the uninfected control group. In the IBV group, embryo mortality began at 3 dpi and reached 71.4% by 4 dpi. PB28 treatment significantly enhanced embryo survival following IBV infection (Figure 6D). Measurement of embryo length showed normal development in the negative control group, with an average length of 9.3 ± 0.3 cm. By contrast, IBV-infected embryos measured 6.3 ± 0.3 cm in length. Embryos receiving low, medium, and high doses of PB28, and ribavirin treatment, exhibited enhanced development, with lengths of 7.1 ± 0.2 cm, 7.2 ± 0.2 cm, 7.9 ± 0.4 cm, and 7.1 ± 0.3 cm, respectively, all significantly greater than those in the IBV group (Figure 6E). Moreover, PB28 treatment reversed the IBV-induced decrease in embryo weight (Figure 6F) and significantly reduced viral titers within the embryos (Figure 6G). These results suggest that PB28 promoted embryonic development during IBV infection in a dose-dependent manner.

### 3.7 Protective efficacy of PB28 against IBV infection in chick models

**Figure 7:**
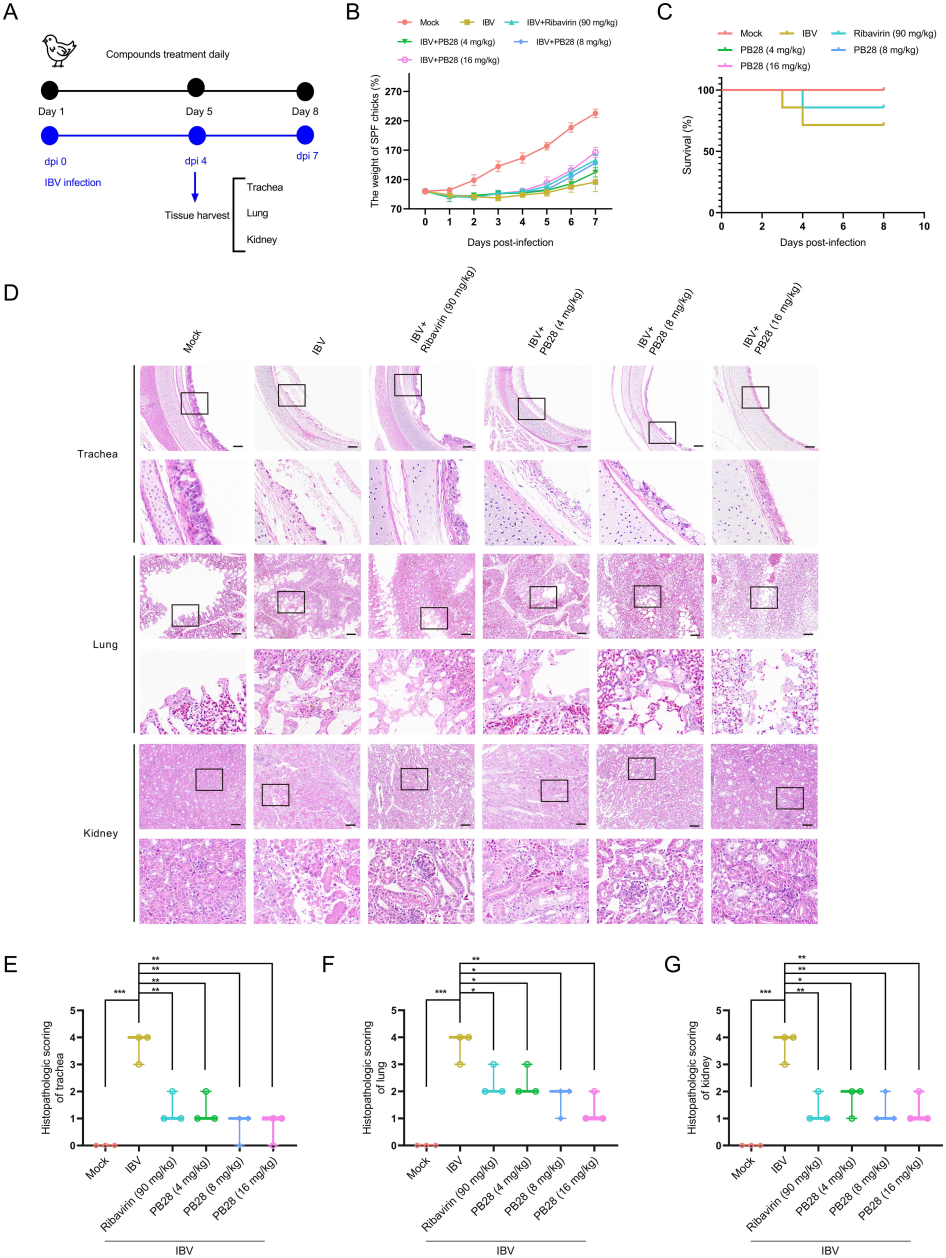
PB28 confers protective efficacy against IBV infection in chicks. **(A)** Schematic diagram of the IBV infection and treatment procedure. **(B)** Body weight of chicks at 7 dpi (*n* = 7). **(C)** Survival curves are recorded over the observation period (*n* = 7). **(D)** Representative H&E staining images of trachea, lungs, and kidneys (*n* = 3; scale bar = 100 μm). **(E–G)** Histopathological scores of the trachea, lungs, and kidneys (*n* = 3). *p < 0.05, **p < 0.01, ***p < 0.001, ****p < 0.0001.

The broad-spectrum antiviral efficacy of PB28 *in vivo* was evaluated using a chick model of IBV infection. The experimental design is illustrated in Figure 7A. PB28 treatment significantly mitigated weight loss in infected chicks (Figure 7B) and enhanced survival rates (Figure 7C). Histopathological analysis of hematoxylin and eosin–stained tracheal, lung, and kidney tissues revealed significant lesions in the IBV-infected group. Lesions included extensive ciliary necrosis and detachment in tracheal epithelial cells, accompanied by abundant serous exudates. In the lungs, inflammatory exudates, capillary congestion, hemorrhage, alveolar wall thickening, and immune cell infiltration in the interstitium were observed. Additionally, kidneys showed tubular epithelial degeneration and necrosis, nuclear pyknosis, and interstitial inflammatory infiltration (Figure 7D). In PB28-treated groups, increasing doses gradually decreased ciliary loss and inflammatory serous exudation (Figure 7D), resulting in lower tracheal histopathological scores (Figure 7E). Consistent with tracheal histopathology, PB28 significantly alleviated IBV-induced lesions in lungs and kidneys (Figure 7E) and reduced histopathological scores in both tissues (Figures 7F,G).

## 4 Discussion

Despite the widespread administration of COVID-19 vaccines, the persistent emergence of immune-evasive SARS-CoV-2 variants and the long-term sequelae of post-acute infection remain formidable global health challenges (Zhang et al., 2023). The development of broad-spectrum antiviral agents, particularly those employing host-targeted strategies, is therefore crucial to addressing current and future coronavirus outbreaks. Although drug repurposing efforts have yielded therapeutic candidates such as remdesivir, the high mutation rate of SARS-CoV-2 facilitates the rapid evolution of drug-resistant strains (Callaway, 2020). Consequently, there is increasing emphasis on interventions that disrupt essential viral replication steps or modulate host cellular processes involved in pathogenesis (Abatematteo et al., 2023). Recent studies have implicated the sigma-1 receptor in SARS-CoV-2 entry and replication, with PB28, a selective sigma-1 receptor antagonist, having exhibited potent antiviral activity *in vitro* (Gordon et al., 2020b). Moreover, silencing or knocking out the sigma-1 receptor markedly reduces SARS-CoV-2 infectivity (Zhang et al., 2023; Gordon et al., 2020a). However, the underlying molecular mechanisms by which PB28 exerts antiviral activity against both emerging SARS-CoV-2 variants and agriculturally important coronaviruses, such as porcine TGEV, PEDV, and avian IBV, remain poorly defined.

In this study, we systematically assessed the antiviral efficacy of PB28 against a broad panel of coronaviruses using both in vitro and *in vivo* models. CCK-8 assays have revealed cell line-dependent variation in the half-maximal cytotoxic concentration (CC_50_) of PB28, potentially attributable to differences in metabolic activity, sigma-1 receptor expression levels, and downstream signaling networks. In Vero E6 cells, PB28 significantly inhibited the replication of SARS-CoV-2, including the Beta, Delta, and Omicron variants. Notably, the antiviral potency of PB28 varied across these variants, possibly due to spike protein mutations that influence ACE2-binding affinity and modulate entry efficiency (Shiehzadegan et al., 2021). Host cell factors such as receptor availability and intracellular viral replication competence may also underlie these differences. Importantly, our results demonstrated that PB28 robustly suppresses the replication of both α-coronaviruses (TGEV and PEDV) and a γ-coronavirus (IBV). This suggests that PB28 may target evolutionarily conserved host pathways essential for coronavirus replication, rather than viral structural proteins that are prone to mutation. Further investigation has demonstrated that knockout of the Sigma-1 receptor markedly suppressed SARS-CoV-2 replication, indicating its essential role as a host factor supporting viral proliferation. Notably, the lack of additive antiviral effects when PB28 was administered in Sigma-1R-KO cells suggests that its antiviral activity is primarily mediated through Sigma-1R antagonism. This supports the specificity of PB28’s mechanism of action and helps rule out off-target contributions. These findings support the further development of sigma-1 receptor antagonists as promising host-targeted broad-spectrum antivirals against both human and animal coronaviruses.

To systematically assess the in vivo antiviral potency of PB28, a series of animal infection models encompassing representative genera of coronaviruses were developed. In K18-hACE2 transgenic mice challenged with the SARS-CoV-2 Delta variant, PB28 exhibited marked antiviral efficacy, as reflected by reduced viral replication, enhanced survival, decreased viral burdens in the lungs, brain, heart, and nasal turbinates, and attenuated tissue pathology. Importantly, while PB28 exhibited antiviral potency comparable to that of remdesivir, it provided greater symptomatic relief and improved survival outcomes. In the BALB/c mouse model of HCoV-OC43 infection, PB28 also exhibited strong antiviral activity, effectively lowering viral burdens and ameliorating virus-induced lesions in the heart, brain, and lungs, with efficacy surpassing that of ARB. Furthermore, in both embryonated egg and chick models challenged with IBV, PB28 markedly enhanced embryo survival, supported embryonic growth, and mitigated virus-induced weight loss and histopathology, demonstrating efficacy comparable to ribavirin. Collectively, these results demonstrate the broad-spectrum antiviral activity of PB28 across distinct coronavirus genera and underscore its therapeutic potential as a candidate agent, offering novel avenues for combating both COVID-19 and avian coronavirus infections.

Due to the high genetic variability of coronaviruses and the limited cross-protection afforded by current vaccines (Li et al., 2019; Lv et al., 2021), coupled with mortality reported in vaccinated poultry (Chen et al., 2019), there is an urgent need to develop novel antivirals with broad-spectrum activity. Our findings demonstrate that PB28 confers antiviral activity comparable to, and in certain preclinical models superior to, remdesivir, ARB, and ribavirin, without eliciting detectable toxicity under experimental conditions. Molecular docking analyses indicate that PB28 selectively binds to the sigma-1 receptor, thereby interfering with virus-host interactions and inhibiting viral replication. These mechanistic insights elucidate the antiviral mode of action of PB28 and provide a rationale for the development of sigma-1 receptor–targeted antiviral agents.

## 5 Conclusion

In summary, our findings demonstrated that PB28 exhibits broad-spectrum antiviral activity against α-, β-, and γ-coronaviruses through a host-targeted mechanism involving competitive binding to the sigma-1 receptor. These findings highlight the therapeutic potential of PB28 against coronavirus infections and support its further advancement toward preclinical and clinical development.

## Data Availability

The original contributions presented in the study are included in the article/Supplementary material, further inquiries can be directed to the corresponding authors.

## Supplementary material

SUPPLEMENTARY FIGURE S1 Analysis of antiviral effects of PB28 on Vero E6 or Sigma-1R-KO cells. *****P* < 0.0001.

SUPPLEMENTARY FIGURE S2 **(A)** Representative H&E staining images of lung, brain, and heart tissues from Delta-infected K18-hACE2 mice treated with PB28 (*n* = 3); scale = 100 μm. **(B–D)** Histopathological scoring of tissues post-PB28 treatment in Delta-infected K18-hACE2 mice (*n* = 3). **P* < 0.05, ***P* < 0.01, ****P* < 0.001, *****P* < 0.0001.
