# Supplementary figures and images for: Broad-spectrum antiviral activity of the sigma-1 receptor antagonist PB28 against coronaviruses
Source: Front Microbiol. 2025 Aug 12;16:1636035. doi: 10.3389/fmicb.2025.1636035 (PMC12378744; doi:10.3389/fmicb.2025.1636035)

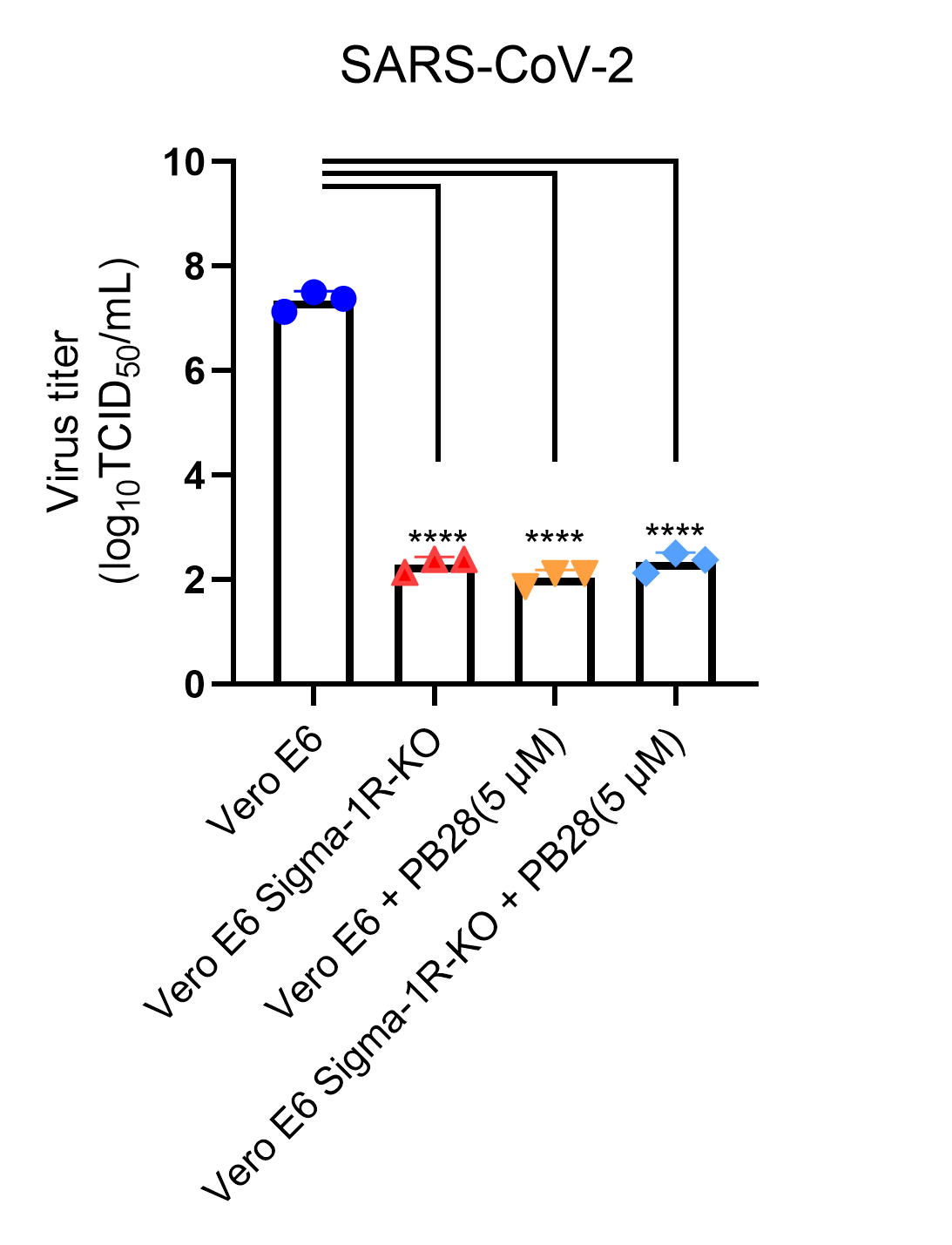

Supplement: SUPPLEMENTARY FIGURE S1 — Analysis of antiviral effects of PB28 on Vero E6 or Sigma-1R-KO cells. ****P < 0.0001. [file Image_1.tif]
