# Supplementary material for: Broad-spectrum antiviral activity of the sigma-1 receptor antagonist PB28 against coronaviruses
Source: Front Microbiol. 2025 Aug 12;16:1636035. doi: 10.3389/fmicb.2025.1636035 (PMC12378744; doi:10.3389/fmicb.2025.1636035)
Supplement: Supplementary file 3 [file Table_1.docx]

**Table 1**

Antiviral Activity, Cytotoxicity, and Selectivity Index of PB28 against Coronaviruses

| **Virus strain** | **Cell line** | **EC_50_ (μM)^a^** | **CC_50_ (μM)^b^** | **SI^c^** |
| --- | --- | --- | --- | --- |
| SARS-CoV-2 | Vero E6 | 1.40±0.01 | 50.64±1.27 | 36.17 |
| Beta Variant | Vero E6 | 2.09±0.22 | 50.64±1.27 | 24.23 |
| Delta Variant | Vero E6 | 2.76±0.06 | 50.64±1.27 | 18.35 |
| BA.1 Variant | Vero E6 | 1.64±0.04 | 50.64±1.27 | 30.88 |
| BA.2 Variant | Vero E6 | 1.46±0.03 | 50.64±1.27 | 34.68 |
| TGEV | ST | 2.01±0.08 | 38.79±1.14 | 19.30 |
| PEDV | Vero E6 | 2.95±0.19 | 50.64±1.27 | 17.17 |
| HCoV-OC43 | HCT-8 | 4.12±0.22 | 63.35±0.82 | 15.38 |
| IBV | Vero E6 | 5.62±0.65 | 50.64±1.27 | 9.01 |

Notes: All data are presented as mean ± standard deviation (n = 3).

a. EC50: the effective concentration of PB28 that provides 50% protection against virus-induced cytopathic effects.

b. CC50: the concentration of PB28 that reduces cell viability by 50%.

c. SI: Selectivity Index (the ratio of CC_50_ / EC_50_).
